# ‘It’s like Taking a Sleeping Pill’: Student Experience of Autonomous Sensory Meridian Response (ASMR) to Promote Health and Mental Wellbeing

**DOI:** 10.3390/ijerph20032337

**Published:** 2023-01-28

**Authors:** Nicole Woods, Julie M. Turner-Cobb

**Affiliations:** Department of Psychology, Bournemouth University, Poole BH12 5BB, UK

**Keywords:** autonomous sensory meridian response (ASMR), mindfulness, mental wellbeing, student health, phenomenological

## Abstract

Autonomous Sensory Meridian Response (ASMR) is purposely elicited by some individuals to promote health and mental wellbeing. The aim of the current study was to explore how ASMR is used and its perceived benefits in a student population. We employed semi-structured qualitative interviews, with eight female students who self-reported as ASMR-sensitive users. Inductive thematic analysis, underpinned by a phenomenological framework, was applied to the data. Two themes, each with three subthemes, were identified; these highlighted the journey from first discovering ASMR to present experience and the use of ASMR to promote health and mental wellbeing both directly and indirectly. For some, ASMR was used daily, whilst for others it was used in a relapsing-remitting fashion: usage increased when struggling with mental wellbeing and was most often used as a tool to induce sleep or distraction when feeling anxious. Participants also reported ASMR-eliciting content as intriguing, and that the phenomenon was regarded as taboo. ASMR appears to play an important role in promoting health and mental wellbeing; frequency of use, preferred triggers, and purpose of use varied, highlighting its flexible and subjective nature. It provides a potential cost-effective tool in populations such as students where mental health needs are burgeoning.

## 1. Introduction

Symptoms of common mental health conditions such as anxiety, low mood and insomnia have increased worldwide in recent years [1,2] with prevalence rates particularly high in student populations [3]. Compared to the general population, higher education students have shown a greater prevalence of poor mental health and increased severity of mental health concerns [3,4,5]. The experience of starting and attending university may create feelings of loneliness and financial concern, as well as academic pressures and worry [6], and university mental wellbeing and counselling services have reported a higher demand on their resources [7].

Given that the COVID-19 pandemic and its related restrictions brought threats to financial stability, food and housing security, and health [8,9] it is unsurprising that the prevalence of mental health problems rose further during 2020/2021 [10]. Within the general population, levels of clinically significant mental distress increased from 18% prior to the pandemic, to 27% in the initial months of the pandemic [11]. Symptoms of anxiety, depression, and stress were observed in 15–33% of individuals within the general population during the initial months of the COVID-19 pandemic [12,13]. During this time, 37.9% of the general population also experienced symptoms of insomnia [14], which may exacerbate poor mental wellbeing and increase the risk of developing chronic health conditions such as cardiovascular disease and type 2 diabetes mellitus [15,16]. With increasing demand, the provision of support has become delayed, with one in four people waiting at least three months for initial assessment, and some up to four years [17].

Popular means to improve mental wellbeing frequently feature mindfulness applications which utilise guided and unguided mindfulness techniques through auditory and visual stimuli [18] and include self-help availability. With over 52 million users across over 2500 different applications [19], individuals report using these applications in order to reduce anxiety and promote sleep [20]. An increasingly used form of stimulus likened to mindfulness [21]) is that of Autonomous Sensory Meridian Response (ASMR). First coined in 2010, ASMR is used to refer to atypical perceptual phenomena when exposed to certain interpersonal, auditory, and visual stimuli [22]. Common audio-visual and interpersonal stimuli which attempt to evoke ASMR are referred to as ‘ASMR triggers’ and include whispering, tapping, and slow hand movements [23]. During ASMR, an individual may experience a pleasant tingling sensation originating at the top of the scalp, which then moves down the neck and potentially to extremities [24]. Frequently accompanied by this perceptual experience is a change in affective state, producing feelings of euphoria and relaxation [25]. The creation and usage of video content which attempts to trigger this response has grown rapidly in popularity [26].

The feeling of relaxation that accompanies ASMR may be a result of physiological changes, although the underlying mechanisms of such changes have been contested [27]. For example, studies measuring the heart rate variability of ASMR-sensitive individuals reveal a reduction in heart rate and blood pressure when watching ASMR content [28,29]. Similarly, the effect of ASMR induced specifically using sound has been found to both increase relaxation and enable mental recovery from challenging cognitive tasks performed in the laboratory [30]. Other work points to the seemingly paradoxical simultaneous co-occurrence of physiological mechanisms of relaxation and activation [31]. In contrast, in an examination of expectancy effects in ASMR-naïve and ASMR-experienced individuals, physiological and affective changes relating to ASMR were found to be due to a placebo effect, but the authors point out that this does not detract from the potential benefits of ASMR [32]. Irrespective of the underlying mechanism, ASMR does appear to cause a reliable change in physiology [33]. Functional MRI studies have observed changes in neural activity whilst watching ASMR-inducing content, with the medial pre-frontal cortex, nucleus accumbens, insula and supplementary motor area being activated and participants experiencing tingling sensations [34,35]. Activation appeared to be less significant when individuals did not experience tingling, suggesting ASMR may be felt on a continuum of sensation intensity [36]. A recent EEG study examining ASMR found increased alpha activity in the parietal and frontal regions of the brain when ASMR-sensitive individuals viewed ASMR-inducing content [21]. These areas of neural activity indicate ASMR may promote feelings of calm through parasympathetic activity [37]. Such physiological changes may include a decrease in heart rate, blood pressure, and respiration rate, which may result in feelings of physical and psychological contentment [38].

Due to changes in physiology and affective state, most individuals who purposely try to evoke ASMR do so to improve mental wellbeing [23]. Some researchers have observed that 86% of participants watch ASMR videos to relax or trigger ASMR, 41% use videos to help them sleep, and 11% to reduce anxiety [39], and others have similarly found that 98% of participants reported watching ASMR to relax, 82% to sleep, 70% to cope with stress, and 80% reported a positive effect on their mood [23]. Whilst previous work has explored why individuals use ASMR through questionnaires [23,39] and anecdotal evidence suggests ASMR may play a role in the maintenance of health and mental wellbeing, a detailed exploration of how individuals use ASMR in this way has not been conducted.

The aim of the current study was to conduct an in-depth exploratory phenomenological examination of ASMR use to promote health and mental wellbeing. In this study, university students were sampled given the known prevalence of mental health symptomatology in this population, to gain insight into how they might attempt to promote their health outside of accessing support services. Semi-structured interviews were utilised with those who regularly watch ASMR-eliciting content. Such an approach was chosen to enable a deeper focus on the individual experience of using ASMR and its perceived benefits for health and wellbeing.

## 2. Materials and Methods

### 2.1. Participants and Recruitment

Psychology undergraduate university students were recruited through opportunistic sampling. Advertisement of the study was placed on the psychology department research recruitment system at the authors’ institution. Each participant received course credit for their participation. Inclusion criteria required participants to have watched ASMR regularly (on average at least once per week) for a minimum of three months, to be at least 18 years of age (no upper age limit), and could be of any gender. 

### 2.2. Procedure and Interview Format

Participants provided informed consent prior to the interview through a Qualtrics survey link and were assured that their confidentiality would be preserved. The online video calling application, Zoom, was utilised to conduct individual semi-structured interviews lasting approximately one hour each. During the interview, participant cameras were turned off and data was audio recorded using the Zoom recording function and stored on the password-protected network of the researchers’ institution. Participants were asked about their experiences of using ASMR and the role it played in their health and wellbeing. The interview schedule was categorised into three subsections of questions to explore: (i) first ASMR use; (ii) current ASMR use; and (iii) the role of ASMR in health and wellbeing (for interview schedule, see Supplementary Material). Subsequent to the interview, each audio recording was transcribed and assigned a pseudonym to ensure participant anonymity. Audio recordings of each interview were then permanently deleted, and data analysis commenced.

One-to-one interviews were selected over focus groups due to the potentially sensitive health topic being discussed, to allow the opportunity for clarification and follow-up [40], and to enable open-ended questions [41]. Semi-structured interviews were selected to allow fundamental questions to be asked whilst providing flexibility for follow-up questions and clarification [42].

### 2.3. Data Analysis

Interview transcripts were analysed using inductive reflexive thematic analysis and underpinned by interpretative phenomenology [43,44]. As a methodological approach, phenomenology seeks to understand phenomena through lived human experience [45] and does not impose a theoretical perspective upon the research [46]. It is particularly useful when exploring complex and ambiguous phenomena [47] and as such was considered the most appropriate underpinning framework for the current study. Reflexive thematic analysis was chosen over interpretative phenomenological analysis (IPA) since the interview schedule covered the breadth of the topic, rather than focussing on one aspect of ASMR in depth. This thematic analytical approach enabled us to capture the heterogeneity and breadth of interviewee responses as recommended [48]. The underlining principles of IPA were still followed and influenced the analysis due to the epistemological freedom that thematic analysis provides [49]. Given this was a relatively small pilot project, we followed sample size guidelines of between 6–10 participants being sufficient for the analytical method employed [50].

Following well-known and established recommendations for thematic analysis [50], the analytical process commenced with familiarisation during transcription of the interviews. Coding was conducted by the first author and then shared and developed through discussion with the second author. Following transcription, a brief summary of each account was noted, along with initial thoughts. Each case was scanned, and initial codes were produced using complete coding. Recurrent codes across all data sets were then highlighted to help identify the main and provisional sub-themes. An initial thematic map was created using these themes, which was reviewed for overlap and revised accordingly. Three themes and eight subthemes were initially coded, which in review identified a strong overlap in two of the three main themes and four of the eight subthemes; these were merged in the final map. The thematic table of final themes was then produced, including examples at the level of each subtheme (see Supplementary Materials for final theme table).

### 2.4. Researcher Self-Reflexivity

As an individual who frequently watches ASMR to promote mental wellbeing, the first author followed an insider approach throughout the process of this research. During interviews, they disclosed their insider perspective to participants in order to make them feel at ease [51], especially when discussing topics perceived as taboo. Moreover, disclosing an insider approach may enhance the rapport and openness of participants [52] resulting in rich responses. Whilst disclosing an insider approach to participants may result in biases [53], it was felt that the potential of this was low and the perceived benefits were high. The first author ensured they remained aware of their insider approach and the influence they may have upon the research throughout to ultimately share the experiences of the interviewees.

### 2.5. Ethical Considerations

Ethical approval was granted by the Department of Psychology Research Ethics Committee of the authors’ institution (#35041) and ethical guidelines outlined by the British Psychological Society were followed. Prior to study commencement, all participants were given a link to a survey through Qualtrics which provided them with a downloadable participant information sheet and online consent form. The information sheet made participants aware of the question content prior to the interview and outlined their ability to withdraw from the study at any point up until the data was anonymised. The information sheet also gave signposting information to local and national mental wellbeing organisations. To assure confidentiality, pseudonyms were used for all participants throughout the interview and only the researcher conducting the interviews had access to the audio files which were permanently deleted post transcription.

## 3. Results

Eight participants were recruited, all of whom self-identified as female, British, and ranged in age from 19 to 52 years. They had all been using ASMR for between 6 months to over 5 years and estimated their use as approximately 3–7 times per week (see Table 1).

**Figure 1:**
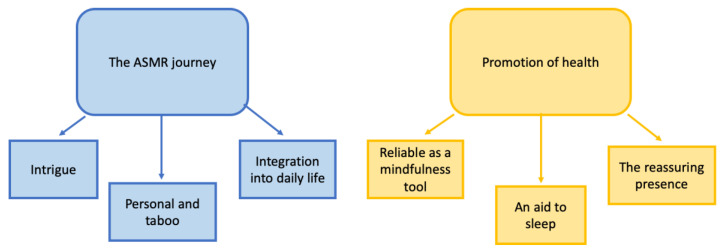
Thematic map outlining themes and sub-themes.

Two themes were identified from the data: (i) the ASMR journey and (ii) promotion of health, each with three subthemes (see Figure 1).

### 3.1. Theme 1: The ASMR Journey

This theme (composed of three subthemes: Intrigue; Personal and Taboo; and Integration into daily life) encapsulates the journey that individuals appeared to take from first discovering ASMR to their present-day experience, how individuals perceived ASMR content initially and why they returned to watch more ASMR inducing content. It also highlights the environment in which ASMR is watched and factors that may influence the frequency of viewing ASMR-eliciting content.

#### 3.1.1. Intrigue

This subtheme highlights that despite recent growth in popularity, ASMR is still considered unusual. There were differences in how individuals first came across ASMR, with some shown content by someone else, ‘I was at my grandparents’ house, and I remember my cousin telling me “Have you heard about this whole ASMR thing? You should give it a listen, like it’s crazy!” and that’s where I found it’ (Clara). Whilst some individuals discovered ASMR through chance, ‘I stumbled across it when it was a trend.’ (Cassie). Differences were apparent in how individuals came across ASMR, yet all showed intrigue around ASMR due to the perception of unusual content, ‘I thought it was interesting and very very different to what I would normally watch or have seen’ (Katie). With such intrigue driving some individuals to continue watching ASMR content, ‘the title interested me, I can’t remember exactly what it said, but it was basically telling me I would have a nice sleep, and that I would wake up positively’.

(Doris)

#### 3.1.2. Personal and Taboo

Due to its unusual content, individuals reported that they felt ASMR was a taboo concept, ‘Yeah. It’s so taboo and it doesn’t need to be… it’s not like it’s something inappropriate, but it does feel like watching porn. It’s not something you could watch with your friends; it feels quite private’ (Emma) and something to be watched alone, ‘I’ve only ever watched it alone’ (Becca). Individuals expressed experiencing self-conscious emotions at watching ASMR content:

No, it sounds weird to say it but it’s like a guilty pleasure. I think I would feel embarrassed or weird watching it with someone else. I don’t even tell my friends that I use it. So, I’ve never watched it with anyone else. Just on my own.

(Emma)

This was linked to the personal nature of viewing ASMR, ‘Yeah definitely, yep. It’s usually at night as well. You know when you’ve done all your day and everything… it wouldn’t be as personal either [watching ASMR with someone else]’ (Doris). However, some individuals reported that ASMR was social in nature, and reported sharing their favourite ASMR videos with friends, ‘I have quite a few friends who use it, so at the time we’d like just send each other links’.

(Alice)

Ultimately, individuals felt that ASMR was something that should be discussed more openly, due to the perceived benefits of the content, ‘I just feel like it should be promoted a bit more in ways to help with health and mental wellbeing because not many people understand it and I feel like there needs to be more information out there’.

(Katie)

#### 3.1.3. Integration into Daily Life

The realisation that ASMR content was beneficial to their health and wellbeing resulted in individuals watching it frequently:

Erm, it wasn’t quite involved, but now I’d say it is involved? It’s become more of a ritual to use to help me go to bed I would have said. Whereas before I wouldn’t use such a thing or wouldn’t have believed it would have had such an impact.

(Cassie)

Participants reported that they had begun to incorporate ASMR as part of their daily lives, especially in night-time rituals, ‘I’ll make myself an Earl Grey tea, put it on my bedside table, get in my jammies, laptop is on the bed and then it’ll be headphones in and out for the count’ (Gemma). Some participants reported feeling dependent on ASMR in order to be able to get to sleep, developing a habit of watching ASMR content every day, ‘Oh, I would say it’s literally like, vital. Because I genuinely could not sleep without it’ (Clara). However, some individuals report watching ASMR less frequently, dependent upon how they felt or what was going on in their lives, ‘when I’ve had a particularly stressful day or I’m handing in, you know, I’ve got an assignment due erm I tend to find that I’ll use the ASMR a lot more, erm, when I feel stressed’ (Gemma). Of note was that individuals reported increased ASMR use when they were having difficulties with sleep:

If I really need to sleep or if I’m quite stressed and I feel like I want to relax and stuff. Um, but yeah, I mainly only watch if like, I know I have to be up at 8:00 AM and it’s 2:00 AM and I really, I’m not in a sleepy mood. I’ll put it on.

(Alice)

Or when struggling with their mental wellbeing, ‘It’s a weird one. It depends on how bad my mental state is, it comes in waves, sometimes I need it every single night for a month. Then sometimes I won’t use it for six months’.

(Emma)

### 3.2. Theme 2: Promotion of Health

This theme highlights how individuals use ASMR eliciting content in order to promote health and mental wellbeing. It also captures how individuals perceive such content to promote their health and mental wellbeing both directly and indirectly. It comprises three subthemes: Reliable as a mindfulness tool; An aid to sleep; and The reassuring presence.

#### 3.2.1. Reliable as a Mindfulness Tool

Individuals reported one of the ways that ASMR promoted their mental wellbeing was by providing a distraction from distressing thoughts:

ASMR is a really good distraction for me… A lot of my anxieties are quite random, but I get quite bad anxiety about like passing away in my sleep or something. So, for me, it’s quite beneficial to have something that will distract me. So, I don’t think about that. And like, I won’t be like monitoring things like my heart rate or stuff like that. I’ll be focused on the video. So that’s why I think stuff like that really helps me in my over thinking.

(Alice)

This allowed them to reduce rumination by focusing on the present, which resulted in feelings of relaxation and even euphoria, ‘I feel like I can just concentrate on that alone, it’s like euphoric. And obviously tingles and complete relaxation’ (Becca). Whilst this feeling of calm occurred at a psychological level, it also appeared to have an effect at a physical level:

I put it on, and it was just like my whole body felt eased? Like it genuinely felt like all the tension in my body just sort of slipped away and I felt relaxed. Exactly like how you feel when you meditate.

(Clara)

Participants reported feeling the tingling sensation characteristic of ASMR, ‘I was instantly relaxed and had the tingly feeling in my back. I was so relaxed by it, even after a minute. I just wanted to listen to it over and over again’ (Becca). However, not all individuals reported experiencing this, ‘erm, I didn’t feel any tingling as such, but it was more about putting my body into this state of calmness’ (Gemma). This suggests that changes in the affective state brought about through ASMR may be differentiated in appraisal as related to specific physical symptoms or a more general relaxation experience.

One participant suggested that whilst ASMR-eliciting content provides a distraction, it requires a lack of distraction too, ‘I do go on other social media platforms like snapchat so I can keep in touch with my friends, so I watch when I’m focussed, and I’ve like ended the conversation with my friends?’ (Cassie), which suggests that watching ASMR content requires a degree of focus and awareness, similar to states of meditation such as mindfulness. Another individual suggested that watching ASMR videos may be beneficial for those trying to regulate their emotions after stressful situations, due to the distraction that ASMR provides, ‘I know some people use them after like panic attacks’ (Alice). However, most individuals reported ASMR was most beneficial to them at night when they found themselves unoccupied and ruminating, which prevented them from being able to sleep:

I need stimulation to sleep because otherwise my brain will take control and I’ll often throw out all the things I have to do tomorrow and then I’ll get stressed or think about the embarrassing thing I did like 10 years ago.

(Alice)

#### 3.2.2. An Aid to Sleep

In this subtheme, we identified that ASMR appears to play a significant role in sleep, with most individuals reporting that they use ASMR primarily to induce sleep, ‘Erm, but just using the ASMR to be able to have a good night’s sleep means that I wake up and I’m in a fresh state of mind, you know?’.

(Gemma)

Whilst some participants highlighted that they had become dependent on ASMR to sleep, ‘I think I’ve got, it’s like a dependency on it now to actually be able to fall asleep’ (Clara), others utilised ASMR for sleep less habitually, mainly at times when they were struggling to sleep, ‘I mainly only watch if like, I know I have to be up at 8:00 AM and it’s 2:00 AM and I really, I’m not in a sleepy mood’ (Alice). One individual highlighted the powerful role that ASMR plays in inducing sleep by comparing it to a sleeping pill, ‘I think I would fall asleep if I watched it during the day. It would be like taking a sleeping pill during the day… it’s like taking a sleeping pill’ .

(Emma)

As students, individuals suggested that through adequate sleep, ASMR indirectly promoted academic success as they woke up feeling more productive, rested, and alert:

I’m more awake and energised in the day to do other things rather than what I wouldn’t have done normally. Like now I’m able to get up earlier, I’m able to engage in conversations better with other people, more awake to do work.

(Cassie)

Consequently, they considered that the use of ASMR improved their affective state:

Erm, it’s just my days… obviously with my mental health it’s had a beneficial impact, but my daily mood is like, I feel… I feel more positive going into each day after having a good night’s sleep and using the ASMR.

(Gemma)

#### 3.2.3. The Reassuring Presence

Some students reported that ASMR provides a reassuring presence, being something they can rely on to work for them, ‘it’s a massive part of my life. It’s turned into a routine. It’s a comfort’ (Doris). However, the type of triggers that individuals watch also contributed to feeling that ASMR was a reassuring personal presence ‘Yeah, I think when they just do like hand movements in front of the cameras and stuff that make it feel like they’re doing it to you’.

(Alice)

The virtual presence of others in ASMR-eliciting videos appeared to enable individuals to feel grounded, especially during the night when they were physically alone and possibly feeling distressed, ‘I think when it’s so late at night and you’re in such a bad state, even though the person isn’t actually there and you’re not actually talking to them, having a voice there talking about something is very reassuring’ (Emma). ASMR was described as a very personal experience, with one student highlighting a genre of ASMR videos that coach viewers through difficult times in life, ‘I know that there are some which are friends talking you through how to overcome situations like break ups and bereavements’.

(Emma)

## 4. Discussion

Experiences in our sample of female-identifying undergraduate students using ASMR were heterogenous and highlighted its personal subjective nature. In this study of how students use ASMR, frequency of use, preferred ASMR triggers, and underlying reasons for using ASMR differed between individuals. However, all interviewees described ASMR as a reliable tool they use to promote health and mental wellbeing; for some, this was by allowing the user to induce sleep, for delayed effects of greater productivity, and a positive effect the subsequent day. For others, ASMR appeared to have more immediate benefits by promoting health through the reduction of anxiety, rumination, and catastrophization.

Many individuals in the current study used ASMR as a sleep aid, which is consistent with previous work [23,39]. Interestingly, one study [39] reported that just 11% of respondents used ASMR to reduce anxiety, compared with half of the interviewees in the current study. Our sample was too small to draw direct descriptive comparisons, yet such differences may reflect the higher prevalence of symptoms relating to anxiety, stress, and distress observed in university students compared to that in the general population [3,4].

The current study confirms earlier findings [23,28,29,34,39] and extends them by providing insight into how ASMR content is perceived. Individuals in our sample reported that they initially perceived ASMR content as intriguing, due to it being unusual. Some participants in the current study also disclosed scepticism of its ability to induce sleep and relaxation, consistent with other work [54]. The intriguing and unusual nature of ASMR content, along with its scepticism, may contribute to the feeling that ASMR is a taboo phenomenon, something reported by several interviewees. This insight into perception of ASMR highlights the potential barriers faced by individuals when accessing content and disclosing viewership to others. Such perceptions are important to consider in future development of ASMR interventions work, in respect to the influence on uptake and adherence [55].

A further insight gained is how individuals use ASMR as a tool for health and mental wellbeing. Previous work also found that individuals use ASMR to reduce anxiety [39] but did not suggest how this is achieved. The current study highlights that ASMR is used as a distraction technique from anxious thoughts and distressing physiological responses when individuals are actively anxious. The descriptions provided concur that the use of ASMR to reduce anxiety is similar to the way some other relaxation techniques operate to reduce the stress response, in particular, the way a mindfulness-based stimulus is used [56,57], employing active engagement to be present and avoid rumination [58]. However, there are distinct differences between ASMR and mindfulness. The practice aspect of mindfulness needed to develop the skill [59] is different in ASMR since participants reported gaining benefits as soon as they started watching ASMR-eliciting content, even when not actively engaged with it. ASMR and mindfulness also diverge with respect to the underlying reason individuals report for their use. Mindfulness is often associated with promoting health [60] and whilst ASMR content does promote mental wellbeing, some individuals sought out content intended to trigger ASMR simply for entertainment purposes.

Frequency of ASMR use was variable, from casual to everyday use, with some individuals reporting being unable to sleep without watching ASMR content, whilst others only watched when it appeared in their social media feed. Similarly, individuals reported experiencing ASMR at differing levels of intensity, consistent with other findings [36]. Throughout participant accounts, there was a lack of the distinctive ‘tingling’ characterised as ASMR [22,27]. Some previous work has suggested individuals may not always experience this [36,61]. Several participants in the current study reported never having experienced the tingling sensation associated with ASMR, yet still regarded themselves as ASMR-sensitive since they experienced both affective and physiological changes after viewing ASMR-eliciting content. This raises an interesting question about what categorises an individual as ASMR-sensitive and what a sensory experience must include in order for it to be considered an ASMR response. We would support a taxonomy that distinguishes two distinct ASMR responses, with and without the presence of tingling. Consistent with prior research examining physiological change during ASMR [28,29], individuals in the current study self-reported feeling a decrease in heart rate and respiration rate when watching ASMR content, and these physiological changes were experienced alongside affective changes such as relaxation and euphoria. These physiological and affective changes were not dependent on the presence of the tingling sensation associated with ASMR.

The present study was subject to several limitations. With respect to methodology and recruitment, our inclusion criteria were broad, imposing a minimum age of 18 years but no upper age limit. This resulted in a sample of 7 participants aged 19–23 years and one aged 52 years who had considerably more experience in ASMR use but described the same reasons for watching ASMR and preferred triggers as the rest of the sample. Similarly, their gender identity and undergraduate student status were consistent with the other participants. Importantly, consideration of themes and subthemes in regard to the experience of this older participant and of the data without their contribution did not change the outcome of our analysis. Future work would benefit from systematic consideration of age-specific categories in regard to the benefit of ASMR. We acknowledge that our sample of eight participants was relatively small and limited to one higher education institution, hence findings might be limited in generalisability. However, this sample size is considered acceptable for a small project utilizing qualitative thematic analysis [50] that acknowledges a subjective phenomenological rather than a positivist realist approach and we enabled reliable information gathering through attention to guidelines and recommendations for this approach. Further research is called for that builds on these preliminary findings, including quantitative testing of the benefits of ASMR use and objective assessments that encompass physiological outcomes assessed over time. Such work could also address questions about mechanisms involved in ASMR, with consideration of individual and sociodemographic differences, and other aspects that might influence its use and perceived benefits, and whether ASMR might have addictive characteristics for some individuals. Given that the sample all identified as female, we can only draw conclusions with respect to female-identifying participants. It is of interest that only female participants chose to sign up for the study, possibly highlighting a gendered aspect of the personal and taboo nature of ASMR. It was our aim in this study to examine the individual experience and, as such, we did not seek to directly evaluate the outcome of ASMR use but relied on retrospective self-reporting and we acknowledge that this is a limitation since reflection is potentially open to bias [62]. The advantage of considering in-depth experience was considered to outweigh this bias but further work is needed that extends to direct outcome evaluation. Finally, we acknowledge that our sample is composed solely of participants who endorsed the use and benefits of ASMR and that important information may be derived from participants for whom ASMR practice may not have been found to be beneficial.

Future work would benefit from utilising ASMR elicitation in an intervention study to directly examine health and mental wellbeing effects and associated physiological outcomes. ASMR may be particularly acceptable, appropriate and applicable to a student population and provide a useful and much-needed tool with which to enhance and promote student health and well-being. Future work should seek to further explore the similarities identified between ASMR and mindfulness, and examine common underlying mechanisms and pathways.

## 5. Conclusions

Our findings suggest that female student users of ASMR construe the practice as having a range of benefits for their health and mental wellbeing. Further work is needed that examines these benefits using directly measurable outcomes before conclusions can be drawn but this work adds to a growing body of findings that suggests ASMR may have considerable potential as an intervention tool in non-clinical populations. We would call for further examination and testing to confirm whether the benefits of ASMR could provide a simple and cost-effective tool in populations such as students where mental health needs are burgeoning and underserved.

## Figures and Tables

**Table 1:** Participant demographic information and characteristics of ASMR use.

Pseudonym	Age (yrs)	Time since First Experience (yrs)	Viewing Frequency (days/wk)	Experience of Tingling	Content Viewing Reason	Preferred Triggers
Doris	52	5+	7	Yes	To promote sleep To promote positive mood	Whispering
Cassie	19	2	5–7	No	To promote sleep	Chewing sounds
Becca	20	6 months	6–7	Yes	To promote sleep	Tapping Whispering
Katie	20	4	3	Yes	To relax Distraction from overwhelming thoughts	Chewing sounds
Clara	19	5	7	No	To promote sleep	Fan sounds Tapping Whispering
Gemma	19	1	3	No	To promote sleep Distraction from overwhelming thoughts	Tapping Whispering
Alice	19	4-5	4	Yes	Distraction from overwhelming thoughts To promote sleep	Personal attention Whispering Watching others apply makeup
Emma	23	3	3	No	Distraction from overwhelming thoughts To promote sleep	Personal attention

## Data Availability

The terms of our ethical agreement with participants do not allow for sharing data in the form of full interview transcripts. However, the interview schedule and a final theme table including an extensive list of example quotes are provided in Supplementary Materials Table S2.

## Supplementary Materials

The following supporting information can be downloaded at: https://www.mdpi.com/article/10.3390/ijerph20032337/s1, Table S1 interview schedule; Table S2 final theme table with subthemes and examples.
