# Supplementary material for: ‘It’s like Taking a Sleeping Pill’: Student Experience of Autonomous Sensory Meridian Response (ASMR) to Promote Health and Mental Wellbeing
Source: Int J Environ Res Public Health. 2023 Jan 28;20(3):2337. doi: 10.3390/ijerph20032337 (PMC9915972; doi:10.3390/ijerph20032337)
Supplement: Supplementary file 1 [file ijerph-20-02337-s001.zip › ijerph-2141599-SI.pdf]

‘It’s like taking a sleeping pill’: Student experiences of using Autonomous Sensory Meridian Response (ASMR) to promote health and mental wellbeing

Supporting Information

Contents

Table S1 Interview schedule Page 2

Table S2 Final theme table with subthemes and examples Page 4

Supplemental Material 1. Interview schedule

Pre-interview:
Check participant is comfortable; confirm they have read the participant information sheet and signed the participant agreement form to give fully informed consent to participate in the study. Due to current circumstances, these forms will be in online format and not signed in person, ensure participants are aware of this. If this is all okay, proceed to introduction of the study.

Introduction
Outline:
- the purpose of the study (to gain insight into experiences of using ASMR, especially in relation to the role it plays in your own health and wellbeing)
- motivation for the study
- reiterate study length (1 hour, although this may vary, as semi-structured approach to interview)

Ethics statement and transition:
I'm going to ask you some questions about your experiences of using ASMR. Whilst the questions are not intended to upset or cause distress, I understand that topics of health and wellbeing may be an upsetting, or uncomfortable topic to talk about. If you want to take a moment, or wish to move on to the next question, please let me know and we can. Equally, if you want to end the interview for any reason let me know. However, after you have taken part in this interview your data will be anonymised, so it might not be possible to identify data. Is this, okay?

Allow chance to ask any final questions before beginning the interview.

Transition: As I have mentioned before, these questions are intended to explore your experiences of ASMR. There are 3 sections of questions, and I will let you know we're halfway through.

Section A – Discovering ASMR
Firstly, I would like to ask you a bit about your first experiences with ASMR
- When did you first hear about ASMR?
- What does ASMR mean to you?
- When did you first seek out ASMR content?
- Why did you first seek out ASMR?
- Tell me about the first time you watched ASMR
- *What happened during this experience, how did you feel, where did you seek out ASMR/how, specific triggers watched, alone? with friends? setting – home/work/school/outside? Did you experience 'tingling' on the scalp? Just relaxation?*

Section B – Current ASMR use
- Why do you continue to watch ASMR?
- How often do you currently view ASMR content?
- When do you watch ASMR? – *certain times of the day? Certain situations only?*
- How do you watch ASMR? *Online? With friends? Alone? What location/setting?*
- What types of triggers do you enjoy? Ie *Fast/slow/inaudible? Tapping/chewing/medical elements*
- What happens when you watch ASMR?
- How does ASMR make you feel?
- Why do you think other people may watch ASMR?

Section C – ASMR, health and wellbeing

This last section is going to explore your experiences of ASMR and the role it plays in your health and wellbeing. *Some of these questions may have been covered in previous question 'why do you use ASMR',*

- What impact has ASMR had on your life?
- Do you use ASMR to promote your wellbeing?
- Do you use ASMR to promote your health?
- How do you feel ASMR affects your physical health? *Why? How does it do this? In what way? Specific example of this?*
- How do you feel ASMR affects your wellbeing? *Why? How does it do this? In what way? Specific example of this?*
- Do you feel anything has changed in your life since starting to watch ASMR?
- How important is ASMR to you? *To your health and wellbeing? To your academic success?*
- Is there anything you'd like to add ASMR on your health?
- Is there anything you'd like to add generally on the topic of ASMR?

Conclude interview.
Thank individual for their time and participating, once again inform of withdraw deadline and remind of who to email for any questions or complaints.

Supporting Information. Final theme table with subthemes and examples.

Theme	Subtheme	Examples
**1. The ASMR Journey**	**1.1 Intrigue**	I stumbled across it when it was a trend. (Cassie).<br><br>Yeah! I thought that was really interesting and there was like a lot of videos about that and how they were eating a box of ice. (Cassie)<br><br>It was more interesting, it didn’t really have much of an effect on me because, mainly because I hadn’t, that would have been the first time I had ever watched or experienced something like that before. I wasn’t really engaging with it properly; I was just watching it for what it was. (Cassie)<br><br>I don’t think I have much else to say other than that sometimes it’s not always for relaxation purposes, it’s just there for a moment of interest because obviously there’s more, erm, there’s more, different types of it available but mainly I just use food ones and that’s more for relaxation purposes for me. (Cassie)<br><br>I was at my grandparents’ house, and I remember my cousin telling me ‘Have you heard about this whole ASMR thing? You should give it a listen, like it’s crazy!’ and that’s where I found it (Clara).<br><br>Yeah, definitely. My flatmates though are like ‘what the hell?’ because I literally blast it and my flatmates are like ‘we can hear fan sounds and it’s really annoying’ and I’m like ‘no it’s not, it’s soothing!’ [laughs] (Clara)<br><br>Erm, I think I think quite a lot of people watch ASMR just because they enjoy it because it makes them, because they just like watching it. Like my best friend I know she watches it just because she enjoys it. She doesn’t watch it for distractions or for like calm, she just watches it because she likes it, she just like the sounds, like the way the sounds make her feel, like you said just tingly, erm yeah, she just likes those sensations. (Clara)<br><br>Yeah, so I was in halls, and I met a girl who’s now my best friend, Amy* and she had issues sleeping and she, I suffer with depression myself and I mentioned that I had issues sleeping and she actually recommended to me that I go and give it a go. Erm, yaknow just plug in your earphones before you go to bed and see if it settles you and yeah, I basically found it from a friend really. (Gemma)

	Yeah, so I came away from her showing me the video and erm, it just was the case of yaknow, if an evening I was struggling to sleep, erm, I would put my earphones in and I would listen, and it would calm me so much it would take me off to sleep. (Gemma)<br><br>The range of whispering helped. I was watching lots of food ones, which changed my thoughts about food in a way? You watch the food ones and then you go away and eat the food that you just watched, and you think those are the sounds I just listened to. It was just a bit weird. (Katie)<br><br>I thought it was interesting and very very different to what I would normally watch or have seen' (Katie).<br><br>I'd say in stressful situations, and procrastination. It was just a relaxing thing to listen to. And it was on social media which made it more popular. (Katie)<br><br>It popped up on my newsfeed in YouTube, and I ignored it for a little bit. It would be on for like three hours. So, I went on one for an hour and I enjoyed it so much, that I went off that one and found an eight hour one. (Doris)<br><br>The title interested me; I can't remember exactly what it said. But it was basically telling me I would have a nice sleep, and that I would wake up positively. I thought "Oh I'll give that a go", and it actually worked. (Doris)<br><br>I remember there being a lot of blue it in, the colour. I was drawn to that. It was very interesting. (Doris)<br><br>But would you get the most benefit from it if you did it with someone else? I guess you'd have to try it, it depends on who you did it with. (Doris)<br><br>Yeah, I can't remember the titles, but they had words like positive in them (Doris)<br><br>The trigger words I mentioned, yeah. If I see those types of trigger words on my feed, then I will go on that video (Doris)<br><br>Close my eyes and relax and let the words flow (Doris)<br><br>Probably the same reason, relaxation, mental health. Probably curiosity (Doris)<br><br>I first heard about ASMR a long time ago. I think it was on Buzzfeed the first time I heard about it? I tried listening to it a couple of times and I didn't really feel anything and found it quite boring. Then about six months ago I was scrolling through TikTok and this woman's ASMR page came up and then I felt so relaxed after listening to it. So, yeah it was

	about six months ago that I started listening to it every day. (Becca)<br><br>Yeah, so it was this woman's advertisement for her YouTube. So, after I had seen this, I started watching her YouTube every night before I went to bed. (Becca)<br><br>Um, I would describe it as, I think they're primarily videos, but I think there are like podcasts as well and other forms of media, which are designed to make the, uh, watching the viewer, um, feel relaxed through, um, triggering, I think… Is it the auto sensory meridian response, I think is the term? Yeah. Yeah. Sometimes I think they're designed to make you feel sleepy other times. I think it's to make you feel relaxed or calm, but I mean, that's the main intention. (Alice)<br><br>Pretty much. I remember, I think it helped me let the first time I was like, oh, I feel a lot more tired and like relaxed now. And then I think I just kept that I'm seeking out. I know that now I only really search for if it's, if like I'm quite desperate to sleep. Yeah. But yeah. (Alice)<br><br>Yeah. I think, I think a lot of people, I know, watch it for sleep reasons. I think one of my friends I'm, I'm convinced that she watches it to seek out the weirdest one. She can find, I think it's become a bit of a challenge for her… there's some strange ones out there, but, um, definitely I think the main one is to relax. (Alice)<br><br>I know some people use them after like panic attacks, but I think as well, the younger generation, like people that are even younger than me, people who are like 15, 16 now, I feel like a lot of them like just watch it for entertainment. Cause I know that I think, I can't remember what magazine it has… but they have a series of celebrities doing it. And I don't think people really watch that for sleep. I think that is more for entertainment. (Alice)<br><br>I think it was probably when I was at college. I was going through a break-up; I was really stressed with my college work, and I think I had just "YouTubed" how to stop overthinking or how to meditate or calm down. Because when I would go to bed, I would overthink everything and I would get myself all distressed and I would stop sleeping. Then an ASMR video came up and that was how I first heard of ASMR and at first it was a bit "what is this?", but then I started using it more religiously. (Emma)<br><br>The first time, it wasn't comfortable. Because like I said, it felt weird, it felt wrong. I don't know why it felt wrong when there's nothing wrong about ASMR. It just felt so

	peculiar to me. But after a couple of minutes, I relaxed I'd stopped thinking about what I was thinking about, you get so distracted by it and involved in it. Then the next thing you know, you're asleep. (Emma)
**1.2 Personal and Taboo**	I don't feel like it would've had a massive effect on me because it would've been the first time, I watched something like that… but I thought it was interesting and very very different to what I would normally watch or have seen on Facebook to then have gone on to YouTube. (Cassie)<br><br>Erm, I'd say now it's more erm, it wasn't quite involved, but now I'd say it is involved? It's become more of a ritual to use to help me go to bed I would have said. Whereas before I wouldn't use such a thing or wouldn't have believed it would have had such an impact. (Cassie)<br><br>But then I got in like a loophole, not a loophole, like a uhm, like a deep hole, of just like finding other ASMRs and trying to find ones that I liked, because she loved these eating ones, and I was like 'I wanna find one that I love!' so I went through all that. (Clara)<br><br>Yes! My best friend like she only goes to sleep listening to that ASMR and I'm like 'how in the world can you sleep with that?' I hate it [laughs]. (Clara)<br><br>Yeah, definitely. My flatmates though are like 'what the hell?' because I literally blast it and my flatmates are like 'we can hear fan sounds and it's really annoying' and I'm like 'no it's not, it's soothing!' [laughs] (Clara)<br><br>Erm, I think I think quite a lot of people watch ASMR just because they enjoy it because it makes them, because they just like watching it. Like my best friend I know she watches it just because she enjoys it. She doesn't watch it for distractions or for like calm, she just watches it because she likes it, she just like the sounds, like the way the sounds make her feel, like you said just tingly, erm yeah, she just likes those sensations. (Clara)<br><br>I briefly did go back to my room, and I think I just stuck on, I can't specifically remember what kind of video it was and who it was, but I think I stuck on one and immediately it, I just couldn't believe how calm I felt? After using it. (Gemma)

	I love like the tapping and the whispering, they're the kind of like, things that I, that I look for when in the videos say I'm going to put one on in the evening (Gemma)<br><br>Erm, I, well, for example my friend Amy* who showed it to me she enjoys like, erm, there's like triggers like, this is going to sound like really weird, but you know the like mouthy like the wet [smacks lips to make sound] sounds, she watches that purely because like…just for satisfaction because she loves it, just to feel satisfied by the noises, whereas I'm more trying to get into it to more change my state of mind if you know what I mean? (Gemma)<br><br>But she purely does it for the enjoyment and the satisfaction of the noises and stuff, whereas I watch it to almost unwind and detach myself from the stressful day I've had. (Gemma)<br><br>The range of whispering helped. I was watching lots of food ones, which changed my thoughts about food in a way? You watch the food ones and then you go away and eat the food that you just watched, and you think those are the sounds I just listened to. It was just a bit weird. (Katie)<br><br>Well, what I'd say about schools, is that it should be used more for students to relax. Teachers should show something like ASMR as a distraction. (Katie)<br><br>I just feel like it should be promoted a bit more in ways to help with health and wellbeing because not many people understand it and I feel like there needs to be more information out there (Katie).<br><br>it could even help as a therapy technique (Katie)<br><br>Yeah definitely, yep. It's usually at night as well. You know when you've done all your day and everything? Then you go to bed and you're scrolling your phone. I'm single, so you scroll through your phone, it's very nice when you fall upon them. Well, I don't fall upon them, I have them all on my newsfeed. I'm always listening to them, it's the last thing I listen to. (Doris)<br><br>It wouldn't be as personal either. (Doris)<br><br>But would you get the most benefit from it if you did it with someone else? I guess you'd have to try it, it depends on who you did it with. (Doris)

	And what I found is there’s a difference between having a shower or bath beforehand… having a bath beforehand made it better. (Doris)<br><br>Yeah. It’s so taboo and it doesn’t need to be... it’s not like it’s something inappropriate, but it does feel like watching porn. It’s not something you could watch with your friends; it feels quite private (Emma)<br><br>I’ve only ever watched it alone. (Becca)<br><br>So, I have to have all the lights off. I use my phone rather than my laptop. I lie on my bed and have it set up in front of me. I know it will put me to sleep so quickly that I need all the lights to be off (Becca)<br><br>You know like nail taping? Or something textured. I love that so much (Becca)<br><br>Yeah, it would have been on social media. I have a feeling, I found it YouTube recommended. Yeah. Um, I think?... I struggled quite a lot…. we've not been able to sleep during that time. That's why I must've clicked on it, but I do… me and my friends...I have quite a few friends who use it. So, at the time we'd like just send each other links, so it could have been through that as well, but yeah. (Alice)<br><br>Um, I would describe it as, I think they're primarily videos, but I think there are like podcasts as well and other forms of media, which are designed to make the, uh, watching the viewer, um, feel relaxed through, um, triggering, I think… Is it the auto sensory meridian response, I think is the term? Yeah. Yeah. Sometimes I think they're designed to make you feel sleepy other times. I think it's to make you feel relaxed or calm, but I mean, that's the main intention. (Alice)<br><br>Yeah. It's not a ritual and it's not something like, I don't necessarily seek out for like enjoyment purposes or entertainment. It's mainly just, just to help (Alice)<br><br>I would say there are some weeks where I won't watch it at all and, and there was some weeks I watch every single night. Um, it is very, very irregular for me (Alice)<br><br>I’m trying to think, which also I enjoy, um, I quite like the personal attention ones and I quite like measuring ones, which is quite a unique one. I think. Um, they're quite

	relaxing to me. I quite like the sound of like makeup brushes and things like in the makeup doing your makeup type videos. Those are, are quite relaxing to me. I think I quite like those, like the repetitive, like whispering sounds. Those are quite… quite nice as well. (Alice)<br><br>Yeah. I think, I think a lot of people, I know, watch it for sleep reasons. I think one of my friends I'm, I'm convinced that she watches it to seek out the weirdest one. She can find, I think it's become a bit of a challenge for her… there's some strange ones out there, but, um, definitely I think the main one is to relax. (Alice)<br><br>I know some people use them after like panic attacks, but I think as well, the younger generation, like people that are even younger than me, people who are like 15, 16 now, I feel like a lot of them like just watch it for entertainment. Cause I know that I think, I can't remember what magazine it has… but they have a series of celebrities doing it. And I don't think people really watch that for sleep. I think that is more for entertainment. (Alice)<br><br>Well, I do see like videos that come up in my YouTube recommended all the time, like Kylie Jenner, unintentional ASMR. So, I do think there is a part like an entertainment aspect now and not just trying to sleep because every time I've watched, like the celebrity ones, I can't really sleep because I'm too busy thinking, 'oh wow, it's got to be tough on a microphone.'… Like it doesn't, it doesn't make me feel tired. (Alice)<br><br>Because like, yeah, I know it's a lot of, there was a period when there was quite a lot of, um, teenagers, young teenagers, like 12 to 14 were making like ASMR channels on YouTube and TikTok. So, I think part of it is the entertainment solely and not actual relaxation (Alice)<br><br>I think it was probably when I was at college. I was going through a break-up; I was really stressed with my college work, and I think I had just "YouTubed" how to stop overthinking or how to meditate or calm down. Because when I would go to bed, I would overthink everything and I would get myself all distressed and I would stop sleeping. Then an ASMR video came up and that was how I first heard of ASMR and at first it was a bit "what is this?", but then I started using it more religiously. (Emma)

	It would be a personal attention one, I think it was probably just a friend talking one. It's just about having another voice to concentrate on that isn't in my own head. (Emma)<br><br>The first time, it wasn't comfortable. Because like I said, it felt weird, it felt wrong. I don't know why it felt wrong when there's nothing wrong about ASMR. It just felt so peculiar to me. But after a couple of minutes, I relaxed I'd stopped thinking about what I was thinking about, you get so distracted by it and involved in it. Then the next thing you know, you're asleep. (Emma)<br><br>Because it works. I stop using it when I'm feeling okay. But If I need it, I use it (Emma)<br><br>No, it sounds weird to say it but it's like a guilty pleasure. I think I would feel embarrassed on weird watching it with someone else. I don't even tell my friends that I use it. So, I've never watched it with anyone else. Just one my own. (Emma)<br><br>Yeah, it's not like it's something inappropriate, but it does feel like watching porn. It's not something you could watch with your friends; it feels quite private (Emma)<br><br>Yeah. It's so taboo and it doesn't need to be (Emma)<br><br>Yeah, personal attention ones. I like the brushing on the mic. I don't like the inaudible ones. I'm not against them, but I need something to concentrate on. Otherwise, it's just like background noise (Emma)<br><br>It will be late at night; I get it up on YouTube. My room will always be dark. It depends on the video, if it's a hair brushing one, I will put it under my pillow, so the light doesn't bother me. But if it's a personal attention one or a roleplay I will prop it up and watch it. I'll fall asleep equally as quickly, but I think the seeing someone face to face, especially if I'm having a bad day, is definitely helpful (Emma)<br><br>Insomnia. I think people who have anxiety and it's not just at night, people who go out in public and then they come back, and they may become distressed, so they could use it for that reason. Maybe people who are grieving, I know that there are some which are friends talking you through how to overcome situations. I feel like there are lots of reasons why

		people could use it, I just think that not enough people know about it. (Emma)<br><br>Yeah, it does help. It’s not as intense as a normal YouTube video. It’s very mellow and it’s like someone talking to you. (Emma)<br><br>It’s definitely helped me with my sleep. There was a time where I was suicidal, I was diagnosed as depressed, but I was struggling with my mood, and it would always be at night. So having that tool when I didn’t have anyone, definitely bought me out of some dark places. (Emma)<br><br>It’s very time dependent. It can be crucial to get me to sleep, or I can just forget it exists. It comes in phases (Emma)
	**1.3 Integration of ASMR in Daily Life**	Erm, I’d say now it’s more erm, it wasn’t quite involved, but now I’d say it is involved? It’s become more of a ritual to use to help me go to bed I would have said. (Cassie)<br><br>But because I now use it quite often, I would say it’s had quite a good impact upon my wellbeing because it’s helped me to sleep better essentially and like… sleep has such a huge effect on someone’s wellbeing and good sleep has helped me like be able to feel more lifted and alive, if that makes sense? (Cassie)<br><br>I don’t think I have much else to say other than that sometimes it’s not always for relaxation purposes, it’s just there for a moment of interest because obviously there’s more, erm, there’s more, different types of it available but mainly I just use food ones and that’s more for relaxation purposes for me. (Cassie)<br><br>Well it’s actually really funny because my cousin showed me it in the day and she was like ‘actually that’s like really fun to fall asleep to, if you ever have trouble sleeping it’d be really fun to fall asleep to’ and I was like ‘ah alright, whatever’ not really that bothered about it and then my grandparents walls are paper thin and I sleep in the bedroom that’s next door to my grandad and I could hear him snoring and I was like ‘oh my god, I’m literally not going to fall asleep’ so I was like ‘you know what, let’s try popping on some ASMR and just seeing if it’ll help me fall asleep’ and it did! It literally put me to sleep like a baby! And ever since I’ve been listening to it like every single night. (Clara)<br><br>Erm, it, you know, I started watching the ones that with like erm, tapping like erm, I think it was like a erm block used

	with little buttons on it and they were like pressing the buttons in and it was like keyboard typing and then maybe tapping on the desk and then I really liked that one and it made me fell asleep, like after a while I couldn't keep listening to that one. (Clara)<br><br>Erm, to fall asleep. I think I've got, it's like a dependency on it now to actually be able to fall asleep, so (Clara)<br><br>Yeah, like every night all night, so like 10 hours a night it's on I guess (Clara)<br><br>It's massively helped me sleep and I think that changes so much because sleep dictates all of your mood like if you don't get enough sleep, you can be tetchy, you can be irritable, and so from it helping me get sleep, it's also helping me be calmer and in touch with my feelings. (Clara)<br><br>Oh, I would say it's literally like, vital. Because I genuinely could not sleep without it. (Clara)<br><br>Yeah, so I came away from her showing me the video and erm, it just was the case of yaknow, if an evening I was struggling to sleep, erm, I would put my earphones in and I would listen, and it would calm me so much it would take me off to sleep. (Gemma)<br><br>Erm I started, I continued to watch it because I do suffer with erm depression and anxiety and do really really struggle to almost like, settle and get myself off to sleep erm and I do have like quite broken sleep as well erm so what I do, what I started doing was just literally putting it on before I went to bed, like going and getting into bed, put my earphones in and it put me into this state of calmness and things like the tapping and whispering, erm, I just, I can't even explain it, it like, it just put me into this complete state of relaxation where I would nod off to sleep naturally. (Gemma)<br><br>Yes, that is definitely me, I'll make myself an Earl Grey tea, put it on my bedside table, get in my jammies, laptop's on the bed and then it'll be headphones in and out for the count [laughs] (Gemma)<br><br>Erm, I think it's had a major impact in the sense of thoughts and like struggling with my depression and struggling with being able to get to sleep, erm it had an effect on every aspect of my life you know… being sleep deprived as well as feeling depressed really really took a toll on me and erm,

	it was draining. I felt drained every day and felt like I never had any energy. Erm, but just using the ASMR to be able to have a good night's sleep means that I wake up and I'm in a fresh state of mind, you know? I'm not carrying the stresses of the day before, erm into the new day. Erm, so yeah, it's… it's had a really beneficial impact on my mental health (Gemma)<br><br>Yes, it means that I can get up and be proactive in the day as well, which you know is also a good… is good because you know when you feel like you're bumming out and you're not doing the work, you know, whether it's sleep deprived or stress and anxiety, erm, it's just puts me in a better frame of mind to complete my activities and I do find myself more productive as well. (Gemma)<br><br>Erm, 100%, especially this year. Erm, I had a difficult year last year like I said and in my original second year however erm, using it more has just meant like I said, being more productive, you know, it's helped my academic performance… not only that but my like, my mental health, my moods, my wellbeing in general has benefitted from it. (Gemma)<br><br>Just in a way that they are focusing on something else. It can them be a technique that they can apply to different situations and life scenarios. (Katie)<br><br>it could even help as a therapy technique (Katie)<br><br>So, it's the colours that attract you to it. But then if you fall asleep and relax and put it away from you and just listen to it, I think it will be different. I think you'll get two different feelings from it. One from just watching it and taking words in secondary. First, you're taking in from sight as you're seeing it and then there's the other bit where you're not watching it and just listening to the words. (Doris)<br><br>Yeah definitely, yep. It's usually at night as well. You know when you've done all your day and everything? Then you go to bed and you're scrolling your phone. I'm single, so you scroll through your phone, it's very nice when you fall upon them. Well, I don't fall upon them, I have them all on my newsfeed. I'm always listening to them, it's the last thing I listen to. (Doris)<br><br>Because it works (Doris)

	That's as simple as it is, because it works, and it makes me feel good. If it's going to wake me up in the morning feeling positive, then I'm not going to stop. (Doris)<br><br>At least four times a week, minimum. (Doris)<br><br>Yeah, I can imagine that. I meditate first thing in the morning as well. When I first started, I could have easily have fallen back asleep. But you have to push past it. Meditation at night is spot on. (Doris)<br><br>Yeah, it's the perfect time (Doris)<br><br>And what I found is there's a difference between having a shower or bath beforehand… having a bath beforehand made it better. (Doris)<br><br>It's helped me cope with university and my access course I did before university. It helps me get in the right frame of mind for study (Doris)<br><br>Very, it's a massive part of my life. It's turned into a routine. It's a comfort (Doris)<br><br>I would notice missing the benefits. (Doris)<br><br>I first heard about ASMR a long time ago. I think it was on Buzzfeed the first time I heard about it? I tried listening to it a couple of times and I didn't really feel anything and found it quite boring. Then about six months ago I was scrolling through TikTok and this woman's ASMR page came up and then I felt so relaxed after listening to it. So, yeah it was about six months ago that I started listening to it every day. (Becca)<br><br>Yeah, that was when I started watching it, like I said, every day (Becca)<br><br>Yeah, so it was this woman's advertisement for her YouTube. So, after I had seen this, I started watching her YouTube every night before I went to bed. (Becca)<br><br>I was instantly relaxed and had the tingly feeling in my back. I was so relaxed by it, even after a minute. I just wanted to listen to it over and over again (Becca)<br><br>It's the only thing that helps me sleep (Becca)

	Yeah. I know that if I'm lying-in bed that all I need to do is watch a video and then I'll be asleep (Becca)<br><br>I would say about five of six times a week (Becca)<br><br>So, I have to have all the lights off. I use my phone rather than my laptop. I lie on my bed and have it set up in front of me. I know it will put me to sleep so quickly that I need all the lights to be off (Becca)<br><br>I would say it's improved my sleep schedule so much (Becca)<br><br>Yeah, I would say so, it was a sure-fire thing that I knew would relax me and get me to sleep in a really stressful and painful situation (Becca)<br><br>I would say I would feel a bit lost without it now (Becca)<br><br>Pretty much. I remember, I think it helped me let the first time I was like, oh, I feel a lot more tired and like relaxed now. And then I think I just kept that I'm seeking out. I know that now I only really search for if it's, if like I'm quite desperate to sleep. Yeah. But yeah. (Alice)<br><br>Um, nowadays I, um, I only really watch like watch and seek out if I'm, if I really need to sleep or if I'm quite stressed and I feel like I want to relax and stuff. Um, but yeah, I mainly only watch if like, I know I have to be up at 8:00 AM and it's 2:00 AM and I really, I'm not in a sleepy mood. I'll put it on. (Alice)<br><br>Yeah. It's not a ritual and it's not something like, I don't necessarily seek out for like enjoyment purposes or entertainment. It's mainly just, just to help (Alice)<br><br>I would say there are some weeks where I won't watch it at all and, and there was some weeks I watch every single night. Um, it is very, very irregular for me (Alice)<br><br>I think it was probably when I was at college. I was going through a break-up; I was really stressed with my college work, and I think I had just "YouTubed" how to stop overthinking or how to meditate or calm down. Because when I would go to bed, I would overthink everything and I would get myself all distressed and I would stop sleeping. Then an ASMR video came up and that was how I first heard of ASMR and at first it was a bit "what is this?", but then I started using it more religiously. (Emma)

		Because it works. I stop using it when I'm feeling okay. But If I need it, I use it (Emma)<br><br>It will be late at night; I get it up on YouTube. My room will always be dark. It depends on the video, if it's a hair brushing one, I will put it under my pillow, so the light doesn't bother me. But if it's a personal attention one or a roleplay I will prop it up and watch it. I'll fall asleep equally as quickly, but I think the seeing someone face to face, especially if I'm having a bad day, is definitely helpful (Emma)<br><br>It's definitely helped me with my sleep. There was a time where I was suicidal, I was diagnosed as depressed, but I was struggling with my mood, and it would always be at night. So having that tool when I didn't have anyone, definitely bought me out of some dark places. (Emma)<br><br>Yeah, I find that if I use it to get myself to sleep, I have a better night's sleep. If I have a good night's sleep then I'm more productive, I'm in a better mood. I find my social skills are better, my concentration is better the next day, so I probably should utilise it more, but I just don't. (Emma)<br><br>It's very time dependent. It can be crucial to get me to sleep, or I can just forget it exists. It comes in phases (Emma)
**2. Promotion of Health**	**2.1 Reliable Tool for Mindfulness**	Oh! Okay, so I would say the main purpose of it is for relaxation (Cassie)<br><br>But yeah, they are a form of… they help me relax in a certain way and I'd say that it's much heavier… there's like a lot more content about it now than there ever was before. (Cassie)<br><br>Mainly for relaxation purposes because erm, especially being a university student, you can easily get quite stressed out, erm, I stick mainly with food ASMR just because that's the only one that I particularly enjoy or like watching. But it is mainly for relaxation because it really helps calms me down at the end of the day after doing like a large amount of work for uni and just like other instances. it's just to help me not be so stressed at the end of the day. (Cassie)<br><br>Erm, I, in a sense I feel like there's a lot of tension that has been released, both physically and mentally, because mentally, erm, well, I mean the fact that I'm only focussing on the video really helps with mental stresses from other

	things that are going on. Erm, but physically, it really helps me to relax and just. It's more, a thing to help me sleep than anything. (Cassie)<br><br>Yeah, especially with any pain that I might be feeling from that day. Like I had the vaccination [Covid-19] not too long ago and it helped me to take my mind off of it. (Cassie)<br><br>Yeah, it was good. I think if I'd had a bigger reaction then maybe the ASMR wouldn't have helped as much but because it wasn't too big of a reaction, I feel like the ASMR has a bigger impact? It easily took my mind off of something that wasn't too bad. (Cassie)<br><br>I think it's just the sense of like, white noise that can calm you and just taps into your subconscious almost to make you focus on something else and like your worries maybe or. For me it's like, in a sense, a form of meditation in a really weird way? (Clara)<br><br>Obviously, I was getting really frustrated because I couldn't sleep because of my grandad and then I put it on, and it was just like my whole body felt eased? Like it genuinely felt like all the tension in my body just sort of slipped away and I felt relaxed. Exactly like how you feel when you meditate, it was really nice, so nice it literally like just knocked me out and put me to sleep. (Clara)<br><br>I don't think I ever have had tingles. I definitely don't have it now; I can't really remember if I had it at the beginning, but I don't think I have. It's more just like, eases my body. (Clara)<br><br>My mind is just like it, it's just completely distracted which is so nice because especially at the moment, everyone's minds are so overactive with thoughts of because of lockdown, like there's not a lot of things to do other than think so when I pop it on right before I go to sleep, my whole mind like eases and then from that my body eases and it's, it just like makes me feel really calm and really relaxed. (Clara).<br><br>Yeah, and just peaceful (Clara)<br><br>It's massively helped me sleep and I think that changes so much because sleep dictates all of your mood like if you don't get enough sleep, you can be tetchy, you can be irritable, and so from it helping me get sleep, it's also helping me be calmer and in touch with my feelings. (Clara)

Erm, it just makes me feel a lot less anxious, like I said when I feel quite high emotion, if I just pop it on and distract myself just for a little but, even just one video it distracts me from that anxiety. (Clara)

Yeah, so I came away from her showing me the video and erm, it just was the case of yaknow, if an evening I was struggling to sleep, erm, I would put my earphones in and I would listen, and it would calm me so much it would take me off to sleep. (Gemma)

Erm, well when you said what do I think it is, answering that it's kind of like uh, kind of like a tool -a calming tool I'd say, like obviously through like you know, engaging with our senses, erm in order to put us into a state of, not arousal, but almost calmness and like, destressing. (Gemma)

I briefly did go back to my room, and I think I just stuck on, I can't specifically remember what kind of video it was and who it was, but I think I stuck on one and immediately it, I just couldn't believe how calm I felt? After using it (Gemma)

It was more like, if I noticed anything it was more my breathing like I was almost concentrating on the sounds so much it almost brought my breathing down to this level. (Gemma)

erm, I didn't feel any tingling as such, but it was more about putting my body into this state of calmness. (Gemma)

Erm I started, I continued to watch it because I do suffer with erm depression and anxiety and do really really struggle to almost like, settle and get myself off to sleep erm and I do have like quite broken sleep as well erm so what I do, what I started doing was just literally putting it on before I went to bed, like going and getting into bed, put my earphones in and it put me into this state of calmness and things like the tapping and whispering, erm, I just, I can't even explain it, it like, it just put me into this complete state of relaxation where I would nod off to sleep naturally. (Gemma)

I'd say up to three times a week like in the evenings like if I'm having a bad night or I've had a particularly stressful day, erm, that's almost when my anxiety and my mental health almost declines when I've had a particularly stressful day or I'm handing in, you know, I've got an assignment

	due erm I tend to find that I'll use the ASMR a lot more, erm, when I feel stressed. (Gemma)<br><br>Yeah, it's the switching off mentally and like you said the physical, like, feeling of like then my muscle relax and everything, it's the switching off my active brain in moments when, like you said, I'm overthinking and I can't go to sleep, it would be putting that in and then, I, I literally feel like, this is going to sound really weird, but it feels like I'm floating above my body? Like I don't, I feel completely in a different state. (Gemma)<br><br>Yeah, 100%, yeah! It's almost like, a mini break from everything? Like on a mini holiday, I know it sounds really silly, but like a mini holiday, just taking that time out. (Gemma)<br><br>But she purely does it for the enjoyment and the satisfaction of the noises and stuff, whereas I watch it to almost unwind and detach myself from the stressful day I've had. (Gemma)<br><br>Erm, I think it's had a major impact in the sense of thoughts and like struggling with my depression and struggling with being able to get to sleep, erm it had an effect on every aspect of my life you know… being sleep deprived as well as feeling depressed really really took a toll on me and erm, it was draining. I felt drained every day and felt like I never had any energy. Erm, but just using the ASMR to be able to have a good night's sleep means that I wake up and I'm in a fresh state of mind, you know? I'm not carrying the stresses of the day before, erm into the new day. Erm, so yeah, it's… it's had a really beneficial impact on my mental health (Gemma)<br><br>Yes, it means that I can get up and be proactive in the day as well, which you know is also a good… is good because you know when you feel like you're bumming out and you're not doing the work, you know, whether it's sleep deprived or stress and anxiety, erm, it's just puts me in a better frame of mind to complete my activities and I do find myself more productive as well. (Gemma)<br><br>Erm, 100%, especially this year. Erm, I had a difficult year last year like I said and in my original second year however erm, using it more has just meant like I said, being more productive, you know, it's helped my academic performance… not only that but my like, my mental health, my moods, my wellbeing in general has benefitted from it. (Gemma)

I find it quite calming in a weird way. I find it interesting in different aspects. I don't really know how to explain. Just very calming and helps to relieve stress, I would say. (Katie)

It felt like meditation, I was so relaxed, and I had my headphones in to focus more. (Katie)

I'd say yes. Just like sitting in bed and feeling relaxed and aware of what's happening. I'm not sure how to explain that. (Katie)

Yeah definitely, my mind was in the zone. I wasn't distracted at all I was really engrossed. (Katie)

I'd say in stressful situations, and procrastination. It was just a relaxing thing to listen to. And it was on social media which made it more popular. (Katie)

Yeah, I would sink into my bed whilst watching something that I'd enjoy. With no distractions. (Katie)

Just in a way that they are focusing on something else. It can them be a technique that they can apply to different situations and life scenarios. (Katie)

it's just helped me for mindfulness and to just think of more relaxation techniques when it comes to exams and stuff and like stressful situations. (Katie)

I feel like it's been a relaxation technique. Just helps stay more focused on academic tasks especially during GCSE, having it as a resource was helpful. (Katie)

it could even help as a therapy technique (Katie)

So, it's the colours that attract you to it. But then if you fall asleep and relax and put it away from you and just listen to it, I think it will be different. I think you'll get two different feelings from it. One from just watching it and taking words in secondary. First, you're taking in from sight as you're seeing it and then there's the other bit where you're not watching it and just listening to the words. (Doris)

Relaxed, definitely relaxed. Positive as well. I remember seeing the blue colour and knowing that blue is like positivity. (Doris)

Oh, I get that anyway when I meditate (Doris)

	Yeah, I’m at that stage anyway and when I close my eyes, I see all the colours (Doris)<br><br>Oh yeah, you just sink into the bed (Doris)<br><br>It’s helped me cope with university and my access course I did before university. It helps me get in the right frame of mind for study (Doris)<br><br>Very, it’s a massive part of my life. It’s turned into a routine. It’s a comfort (Doris)<br><br>I was instantly relaxed and had the tingly feeling in my back. I was so relaxed by it, even after a minute. I just wanted to listen to it over and over again (Becca)<br><br>I feel like I can just concentrate on that alone, it’s like euphoric. And obviously tingles and complete relaxation (Becca)<br><br>Yeah, I would say so. I would also say I use it as a form of meditation as well, especially with the breathing techniques. I find it’s like I’m training my brain (Becca)<br><br>Yeah, I would say so, it was a sure-fire thing that I knew would relax me and get me to sleep in a really stressful and painful situation (Becca)<br><br>Um, I would describe it as, I think they're primarily videos, but I think there are like podcasts as well and other forms of media, which are designed to make the, uh, watching the viewer, um, feel relaxed through, um, triggering, I think… Is it the auto sensory meridian response, I think is the term? Yeah. Yeah. Sometimes I think they're designed to make you feel sleepy other times. I think it's to make you feel relaxed or calm, but I mean, that's the main intention. (Alice)<br><br>Pretty much. I remember, I think it helped me let the first time I was like, oh, I feel a lot more tired and like relaxed now. And then I think I just kept that I'm seeking out. I know that now I only really search for if it's, if like I'm quite desperate to sleep. Yeah. But yeah. (Alice)<br><br>I think I definitely got the tingles. That's quiet, commonly associated with that and I was like, oh, this is me. I think, I don't think I fell asleep, but I think I got very sleepy (Alice)

	Um, nowadays I, um, I only really watch like watch and seek out if I'm, if I really need to sleep or if I'm quite stressed and I feel like I want to relax and stuff. Um, but yeah, I mainly only watch if like, I know I have to be up at 8:00 AM and it's 2:00 AM and I really, I'm not in a sleepy mood. I'll put it on. (Alice)<br><br>Yeah. It's not a ritual and it's not something like, I don't necessarily seek out for like enjoyment purposes or entertainment. It's mainly just, just to help (Alice)<br><br>I would say there are some weeks where I won't watch it at all and, and there was some weeks I watch every single night. Um, it is very, very irregular for me (Alice)<br><br>Um, I just, I still get the tingling thing. I think I do get the tingling feeling a bit less now that I've I think I did go through a phase where I watched it like pretty much every night in about maybe year 11 when I was really stressed for exams, and I wasn't getting much sleep. I pretty much watch that. I think most nights. And I think because of that, I'm now a bit immune to them. I definitely still get very like sleepy and drowsy, which is good (Alice)<br><br>Yeah. Kind of full body relaxation a bit like being in a bath, I guess. (Alice)<br><br>Yeah. Pretty, very relaxed. I think it kind of, I often struggle with, I need where they are quite needed stimulation to sleep because otherwise my brain will take control and I'll often throw out all the things I have to do tomorrow and then I'll get stressed or like the embarrassing thing I did like 10 years ago, I don't worry about sleep. It gives me like a focus, and I think it just makes me feel very like a calm and relaxed. I have something to give my attention to until like I drift off (Alice)<br><br>I think… it does grab your attention, but in a way that it's not demanding, it's easy watching when you do watch it. It's still, it does relax you. So, it's still easier to sleep even though it's commanding attention (Alice)<br><br>Yeah. I think, I think a lot of people, I know, watch it for sleep reasons. I think one of my friends I'm, I'm convinced that she watches it to seek out the weirdest one. She can find, I think it's become a bit of a challenge for her… there's some strange ones out there, but, um, definitely I think the main one is to relax. (Alice)

I know some people use them after like panic attacks, but I think as well, the younger generation, like people that are even younger than me, people who are like 15, 16 now, I feel like a lot of them like just watch it for entertainment. Cause I know that I think, I can't remember what magazine it has… but they have a series of celebrities doing it. And I don't think people really watch that for sleep. I think that is more for entertainment. (Alice)

ASMR is a really good distraction for me. Cause I know that for me, a lot of my anxiety are quite random, but I get quite bad anxiety about like passing away in my sleep or something. So, for me, it's quite beneficial to have something that will distract me. So, I don't think about that. And like, I won't be like monitoring things like my heart rate or stuff like that. I'll be focused on the video. So that's why I think stuff like that really helps me in my over thinking. (Alice)

Yeah. Academic stress and success. Definitely. I've used it just for general, like mental health wellbeing. Like if I'm having a really anxious day or, um, or night particularly because I think if I am to get anxious, it does happen quite a lot at night. Um, and just yeah, for general sleep and then trying to cure insomnia and stuff (Alice)

It’s a little bit of like meditation. Like you can do it once a day for 15 minutes and it doesn't make too much of a big impact, but in like the long run it would, whereas I think ASMR it's, it doesn't make a massive impact, but like the, when you do watch it, you do get a benefit from it still a bit the same way as like other ways of mindfulness? (Alice)

I think it was probably when I was at college. I was going through a break-up; I was really stressed with my college work, and I think I had just “YouTubed” how to stop overthinking or how to meditate or calm down. Because when I would go to bed, I would overthink everything and I would get myself all distressed and I would stop sleeping. Then an ASMR video came up and that was how I first heard of ASMR and at first it was a bit “what is this?”, but then I started using it more religiously. (Emma)

I would describe it as auditory stimulation that not necessarily relaxes you, but that triggers something in you that calms your body if that makes sense. (Emma)

		It would be a personal attention one, I think it was probably just a friend talking one. It’s just about having another voice to concentrate on that isn’t in my own head. (Emma)<br><br>No, I don’t experience the tingling that everyone talks about. I calm down and relax, my breathing calms. But I don’t get the tingling, no. I wish I did, but I don’t (Emma)<br><br>Yeah, if you think about it, it won’t happen (Emma)<br><br>It will be late at night; I get it up on YouTube. My room will always be dark. It depends on the video, if it’s a hair brushing one, I will put it under my pillow, so the light doesn’t bother me. But if it’s a personal attention one or a roleplay I will prop it up and watch it. I’ll fall asleep equally as quickly, but I think the seeing someone face to face, especially if I’m having a bad day, is definitely helpful (Emma)<br><br>Insomnia. I think people who have anxiety and it’s not just at night, people who go out in public and then they come back, and they may become distressed, so they could use it for that reason. Maybe people who are grieving, I know that there are some which are friends talking you through how to overcome situations. I feel like there are lots of reasons why people could use it, I just think that not enough people know about it. (Emma)<br><br>Yeah, it does help. It’s not as intense as a normal YouTube video. It’s very mellow and it’s like someone talking to you. (Emma)<br><br>It’s definitely helped me with my sleep. There was a time where I was suicidal, I was diagnosed as depressed, but I was struggling with my mood, and it would always be at night. So having that tool when I didn’t have anyone, definitely bought me out of some dark places. (Emma)
	**2.2 ASMR as a Sleep Aid**	Erm, but physically, it really helps me to relax and just. It’s more, a thing to help me sleep than anything. (Cassie)<br><br>It’s good for sort of relaxation, but it, I wouldn’t say it’s the main thing to help me relax. It’s just a good thing to help me sleep almost. (Cassie)<br><br>Erm… more the fact that it’s helped to erm… I don’t know it’s had the effect on sleep, because then that’s more the

	reason why I would use it and then that’s helped me, like helped me in the day at night because I’m more awake and energised in the day to do other things rather than what I wouldn’t have done normally. Like now I’m able to get up earlier, I’m able to engage in conversations better with other people, more awake to do work, so erm… whereas before I would have stayed in bed until like, later on in the day, and not have done a lot of work whereas now I’m very energised and keeping up to date with everything… sort of thing. (Cassie)<br><br>Yes, that’s how I would say it has helped because the fact that I’m able to have better sleep has then helped me to focus on work, rather than be sleeping, or sleepy erm... or being fatigued during the day. Whereas now I’m able to complete it, finish it, and then move on to the next thing happy with what I’ve fulfilled in my work? (Cassie)<br><br>Well it’s actually really funny because my cousin showed me it in the day and she was like ‘actually that’s like really fun to fall asleep to, if you ever have trouble sleeping it’d be really fun to fall asleep to’ and I was like ‘ah alright, whatever’ not really that bothered about it and then my grandparents walls are paper thin and I sleep in the bedroom that’s next door to my grandad and I could hear him snoring and I was like ‘oh my god, I’m literally not going to fall asleep’ so I was like ‘you know what, let’s try popping on some ASMR and just seeing if it’ll help me fall asleep’ and it did! It literally put me to sleep like a baby! And ever since I’ve been listening to it like every single night. (Clara)<br><br>Obviously, I was getting really frustrated because I couldn’t sleep because of my grandad and then I put it on, and it was just like my whole body felt eased? Like it genuinely felt like all the tension in my body just sort of slipped away and I felt relaxed. Exactly like how you feel when you meditate, it was really nice, so nice it literally like just knocked me out and put me to sleep. (Clara)<br><br>It’s so nice because I’ve always had trouble sleeping. (Clara)<br><br>Erm, to fall asleep. I think I’ve got, it’s like a dependency on it now to actually be able to fall asleep, so. (Clara)<br><br>Yeah, like every night all night, so like 10 hours a night it’s on I guess (Clara)

My mind is just like it, it's just completely distracted which is so nice because especially at the moment, everyone's minds are so overactive with thoughts of because of lockdown, like there's not a lot of things to do other than think so when I pop it on right before I go to sleep, my whole mind like eases and then from that my body eases and it's, it just like makes me feel really calm and really relaxed. (Clara)

It's massively helped me sleep and I think that changes so much because sleep dictates all of your mood like if you don't get enough sleep, you can be tetchy, you can be irritable, and so from it helping me get sleep, it's also helping me be calmer and in touch with my feelings. (Clara).

Yeah, so I came away from her showing me the video and erm, it just was the case of yaknow, if an evening I was struggling to sleep, erm, I would put my earphones in and I would listen, and it would calm me so much it would take me off to sleep. (Gemma)

Just to send me off really and I mean, it did the job so. (Gemma)

I'd say up to three times a week like in the evenings like if I'm having a bad night or I've had a particularly stressful day, erm, that's almost when my anxiety and my mental health almost declines when I've had a particularly stressful day or I'm handing in, you know, I've got an assignment due erm I tend to find that I'll use the ASMR a lot more, erm, when I feel stressed. (Gemma)

Erm, to be honest with you it, I do, I have only used it only in the evenings that's the only time I've ever used it is literally going to bed because that's when my main like, [sighs] struggle really is, going to bed when I feel stressed. (Gemma)

Yes, that is definitely me, I'll make myself an Earl Grey tea, put it on my bedside table, get in my jammies, laptop is on the bed and then it'll be headphones in and out for the count [laughs] (Gemma)

Yeah, it's the switching off mentally and like you said the physical, like, feeling of like then my muscle relax and everything, it's the switching off my active brain in moments when, like you said, I'm overthinking and I can't

	go to sleep, it would be putting that in and then, I, I literally feel like, this is going to sound really weird… but it feels like I'm floating above my body? Like I don't, I feel completely in a different state. (Gemma)<br><br>Erm, I think it's had a major impact in the sense of thoughts and like struggling with my depression and struggling with being able to get to sleep, erm it had an effect on every aspect of my life you know… being sleep deprived as well as feeling depressed really really took a toll on me and erm, it was draining. I felt drained every day and felt like I never had any energy. Erm, but just using the ASMR to be able to have a good night's sleep means that I wake up and I'm in a fresh state of mind, you know? I'm not carrying the stresses of the day before, erm into the new day. Erm, so yeah, it's… it's had a really beneficial impact on my mental health. (Gemma)<br><br>Erm, it's just my days… obviously with my mental health it's had a beneficial impact, but my daily mood is like, I feel… I feel more positive going into each day after having a good night's sleep and using the ASMR. (Gemma)<br><br>Yes, it means that I can get up and be proactive in the day as well, which you know is also a good… is good because you know when you feel like you're bumming out and you're not doing the work, you know, whether it's sleep deprived or stress and anxiety, erm, it's just puts me in a better frame of mind to complete my activities and I do find myself more productive as well. (Gemma)<br><br>Erm, 100%, especially this year. Erm, I had a difficult year last year like I said and in my original second year however erm, using it more has just meant like I said, being more productive, you know, it's helped my academic performance… not only that but my like, my mental health, my moods, my wellbeing in general has benefitted from it. (Gemma)<br><br>It's the only thing that helps me sleep (Becca)<br><br>Yeah. I know that if I'm lying-in bed that all I need to do is watch a video and then I'll be asleep (Becca)<br><br>Yeah, I think that's why I use it so much (Becca)<br><br>I would say it's improved my sleep schedule so much (Becca)

	I wouldn't say so. But it did help me sleep when I had Covid (Becca)<br><br>Yeah, I would say so, it was a sure-fire thing that I knew would relax me and get me to sleep in a really stressful and painful situation (Becca)<br><br>Probably only my sleeping pattern, especially during lockdown. But now it's a lot better (Becca)<br><br>Pretty much. I remember, I think it helped me let the first time I was like, oh, I feel a lot more tired and like relaxed now. And then I think I just kept that I'm seeking out. I know that now I only really search for if it's, if like I'm quite desperate to sleep. Yeah. But yeah. (Alice)<br><br>Um, nowadays I, um, I only really watch like watch and seek out if I'm, if I really need to sleep or if I'm quite stressed and I feel like I want to relax and stuff. Um, but yeah, I mainly only watch if like, I know I have to be up at 8:00 AM and it's 2:00 AM and I really, I'm not in a sleepy mood. I'll put it on. (Alice)<br><br>Um, I just, I still get the tingling thing. I think I do get the tingling feeling a bit less now that I've I think I did go through a phase where I watched it like pretty much every night in about maybe year 11 when I was really stressed for exams, and I wasn't getting much sleep. I pretty much watch that. I think most nights. And I think because of that, I'm now a bit immune to them. I definitely still get very like sleepy and drowsy, which is good (Alice)<br><br>Yeah. Pretty, very relaxed. I think it kind of, I often struggle with, I need where they are quite needed stimulation to sleep because otherwise my brain will take control and I'll often throw out all the things I have to do tomorrow and then I'll get stressed or like the embarrassing thing I did like 10 years ago, I don't worry about sleep. It gives me like a focus, and I think it just makes me feel very like a calm and relaxed. I have something to give my attention to until like I drift off (Alice)<br><br>ASMR is a really good distraction for me. Cause I know that for me, a lot of my anxiety are quite random, but I get quite bad anxiety about like passing away in my sleep or something. So, for me, it's quite beneficial to have something that will distract me. So, I don't think about that. And like, I won't be like monitoring things like my heart

	rate or stuff like that. I'll be focused on the video. So that's why I think stuff like that really helps me in my over thinking. (Alice)<br><br>Um, I wouldn't say it massively has changed anything because I still struggle with my sleep, but, um, I think it, um, it might have eased some of the issues. And I think at the time where I was watching it quite a lot, it probably did change the fact that I was able, I have like a resource that would allow me to sleep a lot quicker. But, um, yeah, I wouldn't say it's necessarily changed my life. I don't think it's made too much of a dramatic enough impact to do that. But if it was a change, it definitely would be more positive than negative. (Alice)<br><br>Yeah, I would say it's more for mental health, but I can see why people would use it if they were feeling that they're physically, they were feeling quite ill. I know that for me, some maybe I think when I'm sick, I get quite restless and stuff, so I probably have used it when I have been ill. I don't have any chronic pain, but I think it's more mental for me, but I can see the benefits of if you had chronic pain, it would take your mind off of it (Alice)<br><br>I think it was probably when I was at college. I was going through a break-up; I was really stressed with my college work, and I think I had just “YouTubed” how to stop overthinking or how to meditate or calm down. Because when I would go to bed, I would overthink everything and I would get myself all distressed and I would stop sleeping. Then an ASMR video came up and that was how I first heard of ASMR and at first it was a bit “what is this?”, but then I started using it more religiously. (Emma)<br><br>Nah, just at night. I think I would fall asleep if I watched it during the day. It would be like taking a sleeping pill during the day… it’s like taking a sleeping pill (Emma)<br><br>Insomnia. I think people who have anxiety and it’s not just at night, people who go out in public and then they come back, and they may become distressed, so they could use it for that reason. Maybe people who are grieving, I know that there are some which are friends talking you through how to overcome situations. I feel like there are lots of reasons why people could use it, I just think that not enough people know about it. (Emma)<br><br>It’s definitely helped me with my sleep. There was a time where I was suicidal, I was diagnosed as depressed, but I

		was struggling with my mood, and it would always be at night. So having that tool when I didn't have anyone, definitely bought me out of some dark places. (Emma)<br><br>Yeah, I find that if I use it to get myself to sleep, I have a better night's sleep. If I have a good night's sleep then I'm more productive, I'm in a better mood. I find my social skills are better, my concentration is better the next day, so I probably should utilise it more, but I just don't. (Emma)<br><br>Like I said with productivity, I am more productive if I have slept, so getting a good night's sleep from ASMR helps with that. (Emma)
	**2.3 A Reassuring Presence**	Yeah, like every night all night, so like 10 hours a night it's on I guess (Clara)<br><br>My mind is just like it, it's just completely distracted which is so nice because especially at the moment, everyone's minds are so overactive with thoughts of because of lockdown, like there's not a lot of things to do other than think so when I pop it on right before I go to sleep, my whole mind like eases and then from that my body eases and it's, it just like makes me feel really calm and really relaxed (Clara)<br><br>Erm, it just makes me feel a lot less anxious, like I said when I feel quite high emotion, if I just pop it on and distract myself just for a little but, even just one video it distracts me from that anxiety. (Clara)<br><br>Yeah, so I came away from her showing me the video and erm, it just was the case of yaknow, if an evening I was struggling to sleep, erm, I would put my earphones in and I would listen, and it would calm me so much it would take me off to sleep. (Gemma)<br><br>Erm, well when you said what do I think it is, answering that it's kind of like uh, kind of like a tool -a calming tool I'd say, like obviously through like you know, engaging with our senses, erm in order to put us into a state of, not arousal, but almost calmness and like, destressing. (Gemma)<br><br>Erm I started, I continued to watch it because I do suffer with erm depression and anxiety and do really really struggle to almost like, settle and get myself off to sleep erm and I do have like quite broken sleep as well erm so what I do, what I started doing was just literally putting it on before I went to bed, like going and getting into bed, put my

earphones in and it put me into this state of calmness and things like the tapping and whispering, erm, I just, I can't even explain it, it like, it just put me into this complete state of relaxation where I would nod off to sleep naturally. (Gemma)

I'd say up to three times a week like in the evenings like if I'm having a bad night or I've had a particularly stressful day, erm, that's almost when my anxiety and my mental health almost declines when I've had a particularly stressful day or I'm handing in, you know, I've got an assignment due erm I tend to find that I'll use the ASMR a lot more, erm, when I feel stressed. (Gemma)

Yeah, 100%, yeah! It's almost like, a mini break from everything? Like on a mini holiday, I know it sounds really silly, but like a mini holiday, just taking that time out (Gemma)

Erm, I think it's had a major impact in the sense of thoughts and like struggling with my depression and struggling with being able to get to sleep, erm it had an effect on every aspect of my life you know… being sleep deprived as well as feeling depressed really really took a toll on me and erm, it was draining. I felt drained every day and felt like I never had any energy. Erm, but just using the ASMR to be able to have a good night's sleep means that I wake up and I'm in a fresh state of mind, you know? I'm not carrying the stresses of the day before, erm into the new day. Erm, so yeah, it's… it's had a really beneficial impact on my mental health. (Gemma)

It's helped me cope with university and my access course I did before university. It helps me get in the right frame of mind for study (Doris)

Mental, emotional, even spiritual, just every part of me, it goes beyond just emotional (Doris)

Very, it's a massive part of my life. It's turned into a routine. It's a comfort (Doris)

I would notice missing the benefits. (Doris)

It's the only thing that helps me sleep (Becca)

Yeah, I think that's why I use it so much (Becca)

Yeah, I would say so, it was a sure-fire thing that I knew would relax me and get me to sleep in a really stressful and painful situation (Becca)

I would say I would feel a bit lost without it now (Becca)

Yeah. It's not a ritual and it's not something like, I don't necessarily seek out for like enjoyment purposes or entertainment. It's mainly just, just to help (Alice)

I'm trying to think, which also I enjoy, um, I quite like the personal attention ones and I quite like measuring ones, which is quite a unique one. I think. Um, they're quite relaxing to me. I quite like the sound of like makeup brushes and things like in the makeup doing your makeup type videos. Those are, are quite relaxing to me. I think I quite like those, like the repetitive, like whispering sounds. Those are quite nice as well. (Alice)

Yeah. Pretty, very relaxed. I think it kind of, I often struggle with, I need where they are quite needed stimulation to sleep because otherwise my brain will take control and I'll often throw out all the things I have to do tomorrow and then I'll get stressed or like the embarrassing thing I did like 10 years ago, I don't worry about sleep. It gives me like a focus, and I think it just makes me feel very like a calm and relaxed. I have something to give my attention to until like I drift off (Alice)

I think… it does grab your attention, but in a way that it's not demanding, it's easy watching when you do watch it. It's still, it does relax you. So, it's still easier to sleep even though it's commanding attention (Alice)

ASMR is a really good distraction for me. Cause I know that for me, a lot of my anxiety are quite random, but I get quite bad anxiety about like passing away in my sleep or something. So, for me, it's quite beneficial to have something that will distract me. So, I don't think about that. And like, I won't be like monitoring things like my heart rate or stuff like that. I'll be focused on the video. So that's why I think stuff like that really helps me in my over thinking. (Alice)

Yeah, I would say it's more for mental health, but I can see why people would use it if they were feeling that they're physically, they were feeling quite ill. I know that for me, some maybe I think when I'm sick, I get quite restless and

	stuff, so I probably have used it when I have been ill. I don't have any chronic pain, but I think it's more mental for me, but I can see the benefits of if you had chronic pain, it would take your mind off of it (Alice)<br><br>I think it was probably when I was at college. I was going through a break-up; I was really stressed with my college work, and I think I had just "YouTubed" how to stop overthinking or how to meditate or calm down. Because when I would go to bed, I would overthink everything and I would get myself all distressed and I would stop sleeping. Then an ASMR video came up and that was how I first heard of ASMR and at first it was a bit "what is this?", but then I started using it more religiously. (Emma)<br><br>I would describe it as auditory stimulation that not necessarily relaxes you, but that triggers something in you that calms your body, If that makes sense. (Emma)<br><br>It would be a personal attention one, I think it was probably just a friend talking one. It's just about having another voice to concentrate on that isn't in my own head. (Emma)<br><br>It's reassuring as well (Emma)<br><br>I think when it's so late at night and you're in such a bad state. Even though the person isn't actually there and you're not actually talking to them, having a voice there talking about something is very reassuring (Emma)<br><br>Because it works. I stop using it when I'm feeling okay. But If I need it, I use it (Emma)<br><br>Yeah, I know it's there (Emma)<br><br>Yeah, personal attention ones. I like the brushing on the mic. I don't like the inaudible ones. I'm not against them, but I need something to concentrate on. Otherwise, it's just like background noise (Emma)<br><br>It will be late at night; I get it up on YouTube. My room will always be dark. It depends on the video, if it's a hair brushing one, I will put it under my pillow, so the light doesn't bother me. But if it's a personal attention one or a roleplay I will prop it up and watch it. I'll fall asleep equally as quickly, but I think the seeing someone face to face, especially if I'm having a bad day, is definitely helpful (Emma)

	Insomnia. I think people who have anxiety and it's not just at night, people who go out in public and then they come back, and they may become distressed, so they could use it for that reason. Maybe people who are grieving, I know that there are some which are friends talking you through how to overcome situations. I feel like there are lots of reasons why people could use it, I just think that not enough people know about it. (Emma)<br><br>Yeah, it does help. It's not as intense as a normal YouTube video. It's very mellow and it's like someone talking to you. (Emma)<br><br>It's definitely helped me with my sleep. There was a time where I was suicidal, I was diagnosed as depressed, but I was struggling with my mood, and it would always be at night. So having that tool when I didn't have anyone, definitely bought me out of some dark places. (Emma)<br><br>It's very time dependent. It can be crucial to get me to sleep, or I can just forget it exists. It comes in phases (Emma).
